# P-1242. Beta-Lactam Therapeutic Drug Monitoring in the Hematology/Oncology Population

**DOI:** 10.1093/ofid/ofaf695.1434

**Published:** 2026-01-11

**Authors:** Melissa Kerkelis, Christina G Rivera (O'Connor), Erin F Barreto, Sara Ausman, Andrew D Rule, Omar M Abu Saleh, Madiha Fida

**Affiliations:** Mayo Clinic, Rochester, MN; Mayo Clinic, Rochester, MN; Mayo Clinic, Rochester, MN; Mayo Clinic Health System - Eau Claire, Eau Claire, Wisconsin; Mayo Clinic, Rochester, MN; Mayo Clinic, Rochester, MN; Mayo Clinic, Rochester, MN

## Abstract

**Background:**

Beta-lactam pharmacokinetics can be significantly altered in patients with malignancy. Therapeutic drug monitoring (TDM; i.e., drug level testing) may facilitate pharmacokinetic/pharmacodynamic (PK/PD) target attainment, but the data are limited. This study aimed to characterize PK/PD target attainment of anti-pseudomonal beta-lactams in hematology/oncology patients.

**Table 1:**
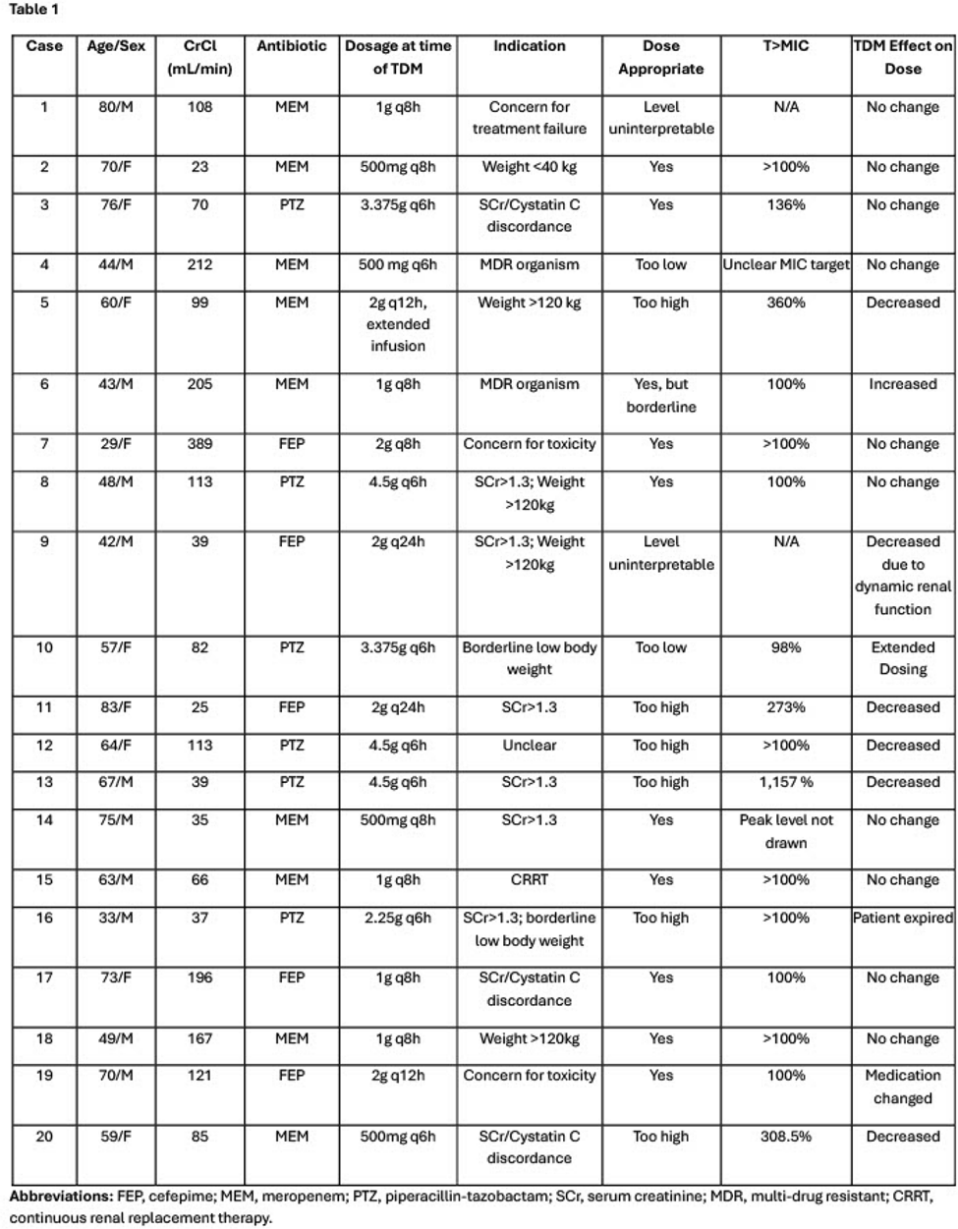
Case Description

**Graph 1:**
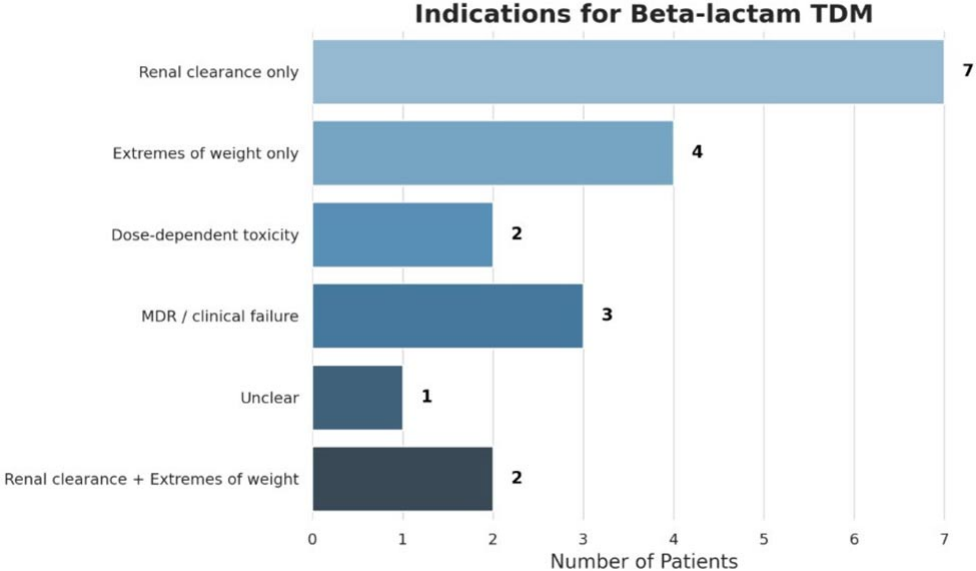
Distribution of Beta-Lactam TDM Indications

**Methods:**

We conducted a retrospective review of beta-lactam TDM performed between June 2022 and June 2024 in hospitalized adults with hematologic or solid organ malignancies. We included patients who underwent beta-lactam TDM during clinical care, typically for extracorporeal membrane oxygenation (ECMO), extremes of body weight, suspected augmented renal clearance, multidrug-resistant infection, or suspected dose-dependent beta-lactam toxicity.

Primary outcomes were (1) the proportion achieving target beta-lactam concentrations and (2) the proportion undergoing antibiotic dose adjustment based on TDM results.

**Table 2:**
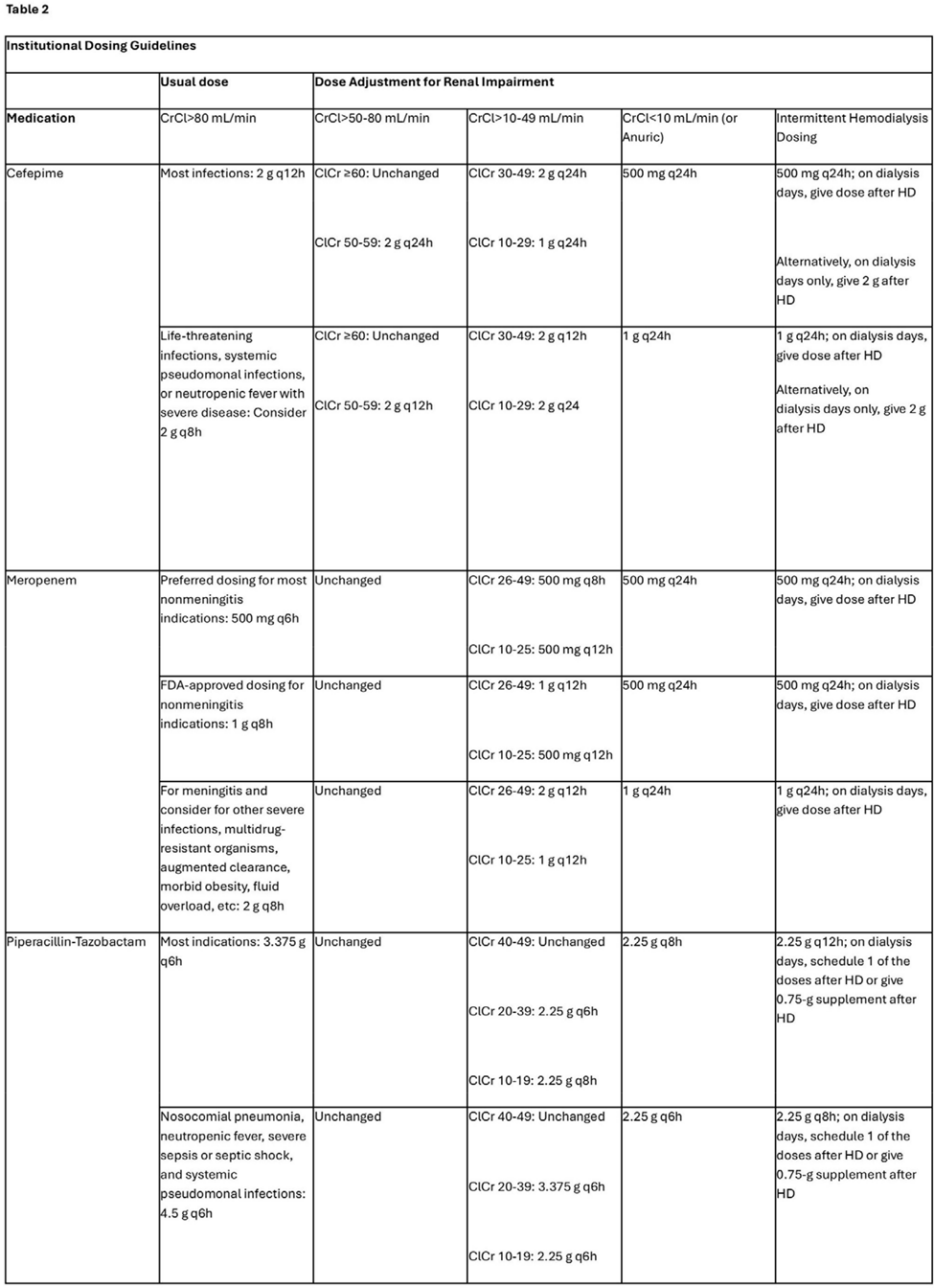
Institutional Dosing Guidelines

**Results:**

There were 47 TDM episodes in 20 unique patients. Considering only the first TDM per patient, 10 (50%) patients achieved target concentrations, 2 (10%) were below target, and 6 (30%) were above target. Antibiotic dose adjustments were made in 8 (40%) patients in response to TDM results. The most common indication for beta-lactam TDM was concerns regarding accurate renal clearance, followed by extremes of weight.

**Conclusion:**

Retrospective findings suggest that beta-lactam TDM facilitated individualization of therapy in 40% of patients with either hematological or solid organ malignancy, with dose adjustments often intended to improve antibiotic safety. Future studies should refine the criteria for beta-lactam TDM use in this population and determine the need for tailored PK/PD targets in patients with impaired host response to infection.

**Disclosures:**

All Authors: No reported disclosures

